# Hand Hygiene Practices in Medical Students: A Follow-Up Study

**DOI:** 10.1155/2014/591879

**Published:** 2014-10-28

**Authors:** Sajad Ahmad Salati, Azzam Al Kadi

**Affiliations:** Department of Surgery, College of Medicine, Qassim University, P.O. Box 6655, Buraidah 51452, Saudi Arabia

## Abstract

*Objective*. The study was conducted to study the impact of various measures instituted to improve hand hygiene practices of the medical students after having documented poor hand hygiene awareness and compliance in a study conducted in 2012. *Methods*. A self-designed questionnaire based on World Health Organization's concept of “Five Moments for Hand Hygiene” was used to evaluate the awareness of the indications of hand hygiene. Compliance was observed during Objective Structured Clinical Examination (OSCE) sessions. Fifty-one students participated voluntarily in the study. *Results*. The awareness and compliance of hand hygiene among the medical students in 2014 had shown statistically significant improvement (*P* < 0.005) as compared to figures of the study conducted in 2012. *Conclusion*. Dedicated multifaceted intervention can improve the hand hygiene practices in medical students.

## 1. Introduction

Hand hygiene is globally acclaimed to be the most cost-effective method for preventing the spread of antimicrobial resistance and reducing healthcare-associated infections (HCAIs) [1]. In a study conducted in 2012, it was found that there were significant deficiencies in the hand hygiene practices of the medical students in Qassim College of Medicine, Saudi Arabia, which needed to be rectified [2]. Various remedial measures were adopted and a follow-up study was conducted from January to March 2014 to study the impact of those measures. The remedial measures included

1. incorporation of hand hygiene in the prescribed syllabus, at various levels;
2. application of perfect hand hygiene practices by teaching faculty, to convey the message by example;
3. incorporation of hand hygiene in examination checklist of OSCE stations;
4. regular feedback/reminders to students erring in hand hygiene practices;
5. dissemination of YouTube links to clips on application of hand hygiene by leading institutions of the world, followed by classroom discussion on these clips;
6. distribution/pasting of posters (at bedside teaching stations) based on the “Five Moments for Hand Hygiene” concept in English and local (Arabic) language;
7. dedication of some faculty members to hand hygiene promotion programme.

## 2. Materials and Methods

A cross-sectional study was undertaken in January-February 2014 in the Department of Surgery of College of Medicine, Qassim University, Saudi Arabia, after having been approved by the research and ethics committee, on the pattern of the study conducted by the department in 2012, where concept of “Five Moments for Hand Hygiene” was adopted for assessing hand hygiene awareness and compliance. Certain activities which are commonly undertaken by medical students during clinical phase (4th year of MBBS course) were selected, and a questionnaire was self-designed for assessing awareness (Table 1).

The purpose of the entire study was explained in very clear terms as per the ethical guidelines of Helsinki, and students were requested to fill the anonymous questionnaires after assuring them of the fact that the results had no impact on their final grades in MBBS examinations. The questionnaire was served to all the enrolled students simultaneously in a single session lasting 15 minutes, towards the end of a routine lecture session. The total number of responses was collected, and data was processed and analyzed using SPSS (statistical package for social sciences, version 11) and Microsoft Excel-97/2000 software.

The compliance of students was assessed by direct observation during OSCE (Objective Structured Clinical Examination) sessions by a board certified surgeon who was involved in neither the drafting of the present survey nor the one which was previously conducted in 2012. This policy was adopted to decrease the possibility of any observer bias. In these OSCE sessions, alcohol hand rub was clearly displayed besides the simulated/actual patients and compliance was seen for four opportunities including

- before general physical examination;
- after general physical examination;
- before lump examination;
- after lump examination.

At the end of the OSCE sessions in March 2014, a feedback was sought from the students to point out the means that were found by them to be most effective in understanding/applying the concept of hand hygiene. This was achieved by requesting the students to respond to a simple anonymous feedback form as shown in Table 2.

## 3. Results

**Figure 1:**
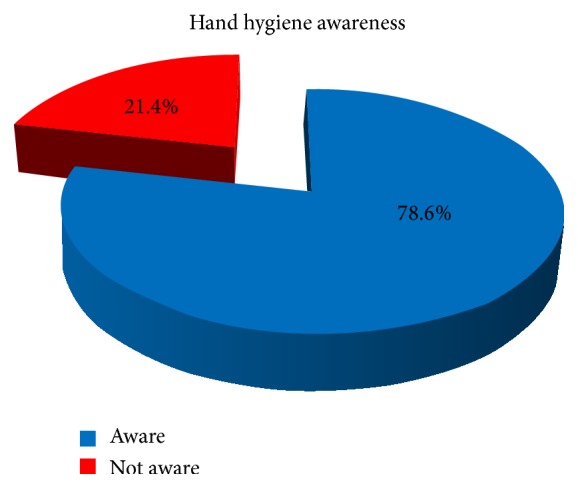
Awareness of medical students regarding moments of hand hygiene.

51 students of 4th year MBBS (34 male and 17 female) participated voluntarily in the study. None of the students refused participation in the study. Each student got five questions in the questionnaire for assessment of awareness about indications of hand hygiene, making a total of 255 questions in 51 students. There were 227 (89%) correct responses and 28 (11%) incorrect/not sure responses (Figure 1). 40 (78.4%) students were able to identify all the five indications of hand hygiene and this percentage was recorded as hand hygiene awareness level. There was no student who identified less than 3 indications. All the errors in responses were concerning the hand hygiene with procedures conducted with gloved hands with 19 errors (7.5%) for hand hygiene before the procedure and 9 (3.5%) errors for hand hygiene after the procedure.

**Figure 2:**
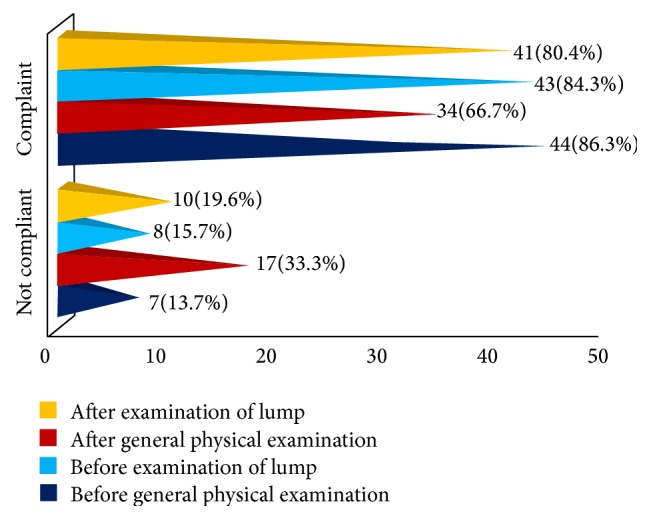
Hand hygiene compliance of the medical students.

As far as the compliance in the present study is concerned, each student got four potential opportunities for hand hygiene during OSCE sessions making a total of 204 opportunities. Hand hygiene was observed at all the four opportunities by 24 students (47%) and this figure was recorded as indicator of hand hygiene compliance. 16 students (31%) missed hand hygiene once, 7 (14%) twice, and 4 (8%) thrice out of the four potential opportunities. None of the students failed to observe hygiene at all the four sites. The hand hygiene compliance profile is depicted in Figure 2.

**Figure 3:**
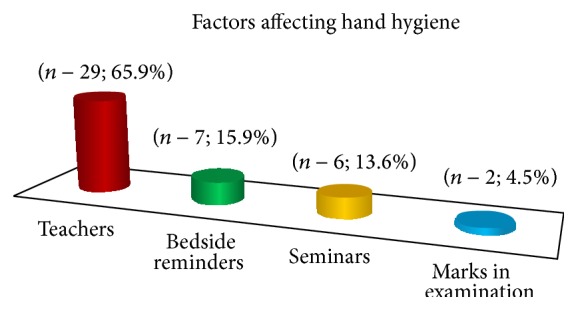
Factor influencing hand hygiene practices of the medical students.

The feedback form was answered by 44 out of 51 students (86.3%). On the basis of that feedback, it was found that 65.9% (29 out of 44) of the students felt the behaviour of the teachers to be the most important factor in motivating them to adopt hand hygiene practices as shown in Figure 3.

## 4. Discussion

**Figure 4:**
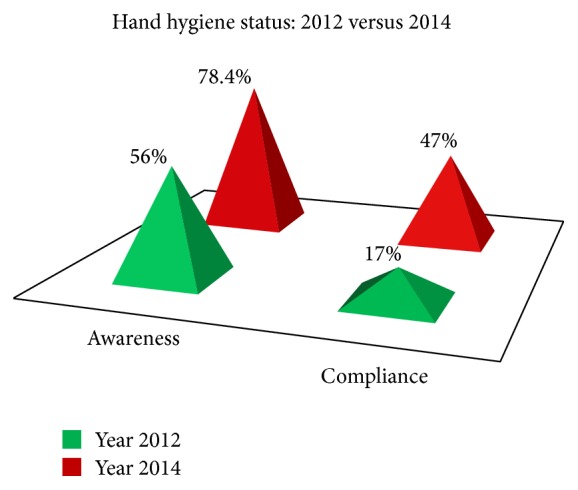
Hand hygiene status of medical students in 2012 and 2014.

Healthcare-associated infection is a very important health issue globally, and proper hand hygiene is highly cost-effective method of infection control [1, 3]. But in spite of simplicity, the compliance is found to be low and an important reason is faulty behaviour development during the phases of training of healthcare professionals [4, 5]. In a study conducted in our college in 2012, the awareness and compliance of hand hygiene in medical students was found to be very low [2] and hence various remedial measures as mentioned in the introduction were taken. As is evident from the results shown in Figure 4, the measures had a statistically significant impact raising the figures of awareness level from 56% to 78.4% and the compliance level from mere 17% to 47%.

The review of recent literature shows that the positive results are achievable both at the level of an institution or on a larger scale. Pittet et al. in a landmark study in 2000 found the dedicated interventions result in sustained improvement in compliance with hand hygiene, coinciding with a reduction of nosocomial infections and MRSA transmission [6]. At an institution level, Fuller et al. in 2012 showed that, in spite of difficulties in implementation, intervention coupling feedback to personalised action planning produced moderate but statistically significant and sustained improvements in hand hygiene compliance [7]. Mestre et al. in 2012 published the positive impact of multifaceted interventions based on WHO and continuous quality improvement methodology [8]. Reichardt et al. in 2013 [9] presented the results of the larger scale campaign, AKTION Saubere Hände, which started in Germany to improve hand hygiene compliance on January 1, 2008. The campaign is designed as a multimodal campaign based on the WHO implementation strategy and funded by the German Ministry of Health and involved more than 700 healthcare institutions, among which are 28 university hospitals. Reichardt et al. found that hand hygiene compliance showed statistically significant improvement as a result of prolonged campaign at all the involved institutions. Even in low-income group countries like Mali (Africa), the interventions have proved to be effective as was shown in a study by Allegranzi et al. in 2010 [10].

As is evident from Figure 3, the hand hygiene practices of the teachers were found to be the most important behaviour modifying factor in students. This feedback of our students corroborates with the many other studies in literature. Snow et al. in 2006 conducted studies on hand hygiene practices of medical students and concluded that the mentor's practice of hand hygiene was the strongest predictor of the student's hand hygiene compliance [11]. Lankford et al. in 2003 compared the hand hygiene compliance in two hospitals and found the compliance to hand hygiene to be influenced significantly by the behaviour of senior healthcare workers [12]. So there is a strong need of attitude modification of teaching faculty with respect to hand hygiene practices so that they can lead by example and pass on the best of the traits to healthcare professionals/mentors of the future.

The errors in responses to questionnaire demonstrated deficiencies in awareness regarding hygiene in activities involving donning of sterile gloves. A similar trend was seen in our previous study conducted in 2012 [2] and hence there is a need to improve the information in students regarding hand hygiene before and after donning the sterile gloves. This can be done by incorporating more of such activities in syllabi where teachers would practically demonstrate the hand hygiene before and after procedures with gloved hands. The issue of errors in hand hygiene in activities accomplished with gloved hands has been projected by many other workers. Fuller et al. in 2011 published the results of an observational study wherein it was concluded that the rate of hand hygiene compliance was significantly lower when gloves were worn and it was stressed that the hand hygiene campaigns should consider placing greater emphasis on the World Health Organization indications for gloving and associated hand hygiene guidelines [13]. Girou et al. in 2004 found that the improper use of gloves limits compliance to hand hygiene and exposes patients to infection [14]. Loveday et al. in a recent study also found the faulty use of gloves to be associated with increased risk of cross-contamination and stressed explicit integration of gloves into hand hygiene policy [15]. Some novel ideas like the use of emollient impregnated gloves [16] have also been mentioned in literature to circumvent the negative impact of faulty usage of hand gloves on hand hygiene compliance. As is evident from results of our present study, the incidence of noncompliance with hand hygiene (*n* − 27) after examination was more than before examination of the patient (*n* − 15). To overcome this type of trend, certain novel innovations like usage of hand sanitizer-dispensing door handles have been advocated in some recent pilot studies [17].

Observation/audit of students during clinical postings for compliance of hand hygiene is an important aspect of implementation of hand hygiene curriculum and development of ideal behaviour. Various different options are mentioned in literature. Direct observation by the faculty member and proper feedback are the policy applied in our department currently. However, review of recent literature shows that engagement and empowerment of the patient to report on hand hygiene compliance are efficient and acceptable to patients and providers, and the results of the observations represent the actual hand hygiene behaviour of the provider [18, 19].

Bedside reminders in form of posters and alcohol hand rub were mentioned by seven students (Figure 3) as having positive impact on hand hygiene. Pittet et al. [6] had also found the promotion of bedside, antiseptic hand rubs to contribute significantly to the increase in compliance by serving as a timely reminder. Electronic monitoring and voice prompts [20, 21] have been mentioned in recent literature and both physical and auditory reminders have been found to significantly improve hand hygiene in healthcare facilities.

Hand hygiene involves behavioural changes [22] and training phase is the best period for achieving perfect practices [4]. The institutions should devise concrete and practical strategies to measure, monitor, and ultimately increase hand hygiene compliance [23]. While devising the strategies, the impact of religious faith and cultural specificities must be taken into due consideration [24]. Once the targets are achieved, there should be continuous monitoring for maintenance of correct hand hygiene practices as there are instances mentioned in literature where change in policies/team leadership reversed the gains [25].

The apparent weakness of our study is the possibility of bias on two fronts.

1. The present study was conducted by the same researchers who had conducted the study in 2012 and had proposed the remedial measures. However, an attempt had been made to decrease observer bias, by taking help from a staff of a different medical school in assessment of compliance. That staff has joined the medical school in 2013 and had no relation with the previous study.
2. The students were briefed about the hand hygiene project and that briefing has chances of altering the responses and hence skewing the results.

The present batch of students is expected to join residency programmes in 2016 and it is planned to conduct a study to assess their in-service hand hygiene practices and compare with hand hygiene practices of other healthcare workers. That study is planned to be conducted in collaboration with designated infection control staff, without any prior information to the students/researchers of the present study, and is expected to provide true insight into the impact of our training programme on hand hygiene behaviour of the students.

## 5. Conclusion

Hand hygiene is a corner stone of infection control strategies and it is important to impart proper hand hygiene education. Dedicated multifaceted interventions can improve the hand hygiene compliance in medical students.

## Figures and Tables

**Table 1:** Self-designed questionnaire used for assessment of hand hygiene awareness.

Is hand hygiene (washing with antiseptic soap/alcohol hand rub) recommended?	Yes	No	Not sure
(1) Before examination of a benign breast lump			
(2) Before donning gloves for blood sugar analysis with glucometer			
(3) After blood sugar analysis (with glucometer) with gloved hands			
(4) After examination of a benign breast lump			
(5) After reading the bedside notes of the patients (without touching the patient)			

**Table 2:** Students' feedback.

Please identify the factor that influenced your hand hygiene practices the most	
Hand hygiene practices of the teachers	
YouTube clips on hand hygiene	
Power-point presentations on hand hygiene	
Posters on hand hygiene kept at bedside training stations	
Fear of losing marks in exam for faulty hand hygiene	
Any other factor	
Any suggestions?	
